# Network Analysis to Identify Communities Among Multiple Exposure Biomarkers Measured at Birth in Three Flemish General Population Samples

**DOI:** 10.3389/fpubh.2021.590038

**Published:** 2021-02-10

**Authors:** Ilse Ottenbros, Eva Govarts, Erik Lebret, Roel Vermeulen, Greet Schoeters, Jelle Vlaanderen

**Affiliations:** ^1^Center for Sustainability, Environment and Health, National Institute for Public Health and the Environment (RIVM), Bilthoven, Netherlands; ^2^Institute for Risk Assessment Sciences, Utrecht University, Utrecht, Netherlands; ^3^VITO Health, Flemish Institute for Technological Research (VITO), Mol, Belgium; ^4^Department of Biomedical Sciences, University of Antwerp, Antwerp, Belgium

**Keywords:** network analysis, human biomonitoring, multiple exposure biomarkers, mixtures, risk assessment, community detection

## Abstract

**Introduction:** Humans are exposed to multiple environmental chemicals via different sources resulting in complex real-life exposure patterns. Insight into these patterns is important for applications such as linkage to health effects and (mixture) risk assessment. By providing internal exposure levels of (metabolites of) chemicals, biomonitoring studies can provide snapshots of exposure patterns and factors that drive them. Presentation of biomonitoring data in networks facilitates the detection of such exposure patterns and allows for the systematic comparison of observed exposure patterns between datasets and strata within datasets.

**Methods:** We demonstrate the use of network techniques in human biomonitoring data from cord blood samples collected in three campaigns of the Flemish Environment and Health Studies (FLEHS) (sampling years resp. 2002–2004, 2008–2009, and 2013–2014). Measured biomarkers were multiple organochlorine compounds, PFAS and metals. Comparative network analysis (CNA) was conducted to systematically compare networks between sampling campaigns, smoking status during pregnancy, and maternal pre-pregnancy BMI.

**Results:** Network techniques offered an intuitive approach to visualize complex correlation structures within human biomonitoring data. The identification of groups of highly connected biomarkers, “communities,” within these networks highlighted which biomarkers should be considered collectively in the analysis and interpretation of epidemiological studies or in the design of toxicological mixture studies. Network analyses demonstrated in our example to which extent biomarker networks and its communities changed across the sampling campaigns, smoking status during pregnancy, and maternal pre-pregnancy BMI.

**Conclusion:** Network analysis is a data-driven and intuitive screening method when dealing with multiple exposure biomarkers, which can easily be upscaled to high dimensional HBM datasets, and can inform mixture risk assessment approaches.

## Introduction

Throughout their life-time, humans are exposed to a plethora of environmental stressors and chemicals that independently or in interaction may have an impact on health. Whereas, chemical risk assessment typically evaluates single compounds, it generally does not appropriately reflect the complexity of concomitant exposure to multiple chemicals in real life. Currently there is yet little insight into commonly occurring exposure mixtures and how these mixtures change between important covariates, e.g., gender, countries, and time. Human biomonitoring (HBM) has the potential to provide a snapshot of exposure to chemicals (1), and these data can be used to screen for the presence of clusters of correlated exposures. The identification of these communities is important for the analysis and interpretation within epidemiological studies (which compounds are more related, and should therefore be considered collectively) and for the design of mixture toxicology studies (which combined exposures do occur in the population), thereby informing risk assessors/managers on potential concomitant exposure pathways.

Patterns between multiple biomarkers are not commonly presented (2). Increasingly, graphical representation of (partial) correlation patterns such as heatmaps or circular correlation globes (circos plots) are being used. However, here the distinction of groups of correlated compounds is not always straightforward as it depends largely on a-priori ordering by the presenter and on the visual interpretation by the reader. Also, the comparison of multiple circos plots [for example as presented in (3)], is challenging, especially when comparing three or more plots or in high dimensional settings. Networks provide a graphical method to represent groups or communities in the data, which has been used widely in the OMICs world (4–6). Applied to HBM data, networks consist of nodes which represent the biomarkers, and edges that represent the conditional dependence between the biomarkers. Networks give an intuitive interpretation of patterns in the data without prior assumptions (7). A network may consist of multiple subnetworks (connected nodes). Within a subnetwork, one or more communities of biomarkers can be detected using community detection algorithms (8). Communities are groups in which nodes (i.e., biomarkers) are more connected to each other than to the rest of the (sub)network. Communities in exposure biomarker networks might therefore represent common exposure routes (dermal, inhalation or ingestion), external sources (such as lifestyle, social or environmental factors) and/or (bio)chemical properties (e.g., kinetics, distribution).

Further insights can be generated with comparative network analysis (CNA), which is an analytical procedure that allows for the comparison of two or more networks based on (dis)similarities (9–11). Comparative network analysis can be used to assess the impact of covariates on observed networks. Differences between networks are presented as (dis)similar nodes and edges, which in itself are amendable to community detection as well (12).

To pilot and illustrate the use of network techniques in exposure HBM data we applied this methodology to data collected as part of the FLEHS (Flemish Environment and Health Study) newborn campaigns (13). The FLEHS data consists of multiple biomarkers, obtained by targeted analysis of cord blood samples collected directly after birth in three subsequent campaigns over a 12 year period (13–17). Time trends of multiple biomarkers across the subsequent FLEHS newborn campaigns (Persistent Organic Pollutants (POPs) and metals) have been described before, showing varying rates of decline of different biomarker over the three campaigns (16).

We were particularly interested in the use of network techniques to visualize biomarker correlation patterns within each FLEHS campaign. In addition, we explored the stability of these networks across sampling campaigns, smoking status during pregnancy, and maternal pre-pregnancy BMI using CNA.

## Materials and Methods

### Flemish Environment and Health Study

In the newborn campaigns of FLEHS, cord blood samples have been collected at three points in time, FLEHS I (*N* = 1,196): 2002-2004, FLEHS II (*N* = 255): 2008-2009 and FLEHS III (*N* = 281): 2013-2014. The FLEHS campaigns are conducted in a population sample that is representative for the geographical distribution and the population density of the population in Flanders, Belgium. A summary of the characteristics of each campaign, including the *p*-value, is presented in Supplementary Table 1. Details of recruitment, sampling, laboratories, limits of detection and quality control measures have been reported before (13, 18, 19). Selection of the chemicals was based on health and exposure related criteria, and technical criteria, extensively discussed by experts as part of the biomonitoring studies (16). The biomonitoring studies were approved by the Ethical Committee of the University of Antwerp (FLEHS I and II) and of the University hospital of Antwerp (FLEHS III).

#### Biomarkers

Chemicals measured in cord blood of newborns were included for analysis if more than 60% of the measurements was above the Limit of Detection (LOD). In FLEHS I, seven biomarkers fulfilled this requirement: cadmium, lead, *p,p*′-DDE, HCB, PCB138, PCB153 and PCB180. In FLEHS II, 12 biomarkers: cadmium, lead, *p,p*′-DDE, PCB138, PCB153 and PCB180, arsenic, copper, manganese, thallium, PFOS, and PFOA. In FLEHS III, 19 biomarkers fulfilled this requirement: all from FLEHS II plus the additional biomarkers: HCB, PCB118, PCB146, PCB170, PCB180, PFHXS, and PFNA. For the CNA comparisons between the three campaigns six corresponding biomarkers were included, and between FLEHS II and III 12 corresponding biomarkers.

#### Imputations and Data Preparation

**Figure 1:**
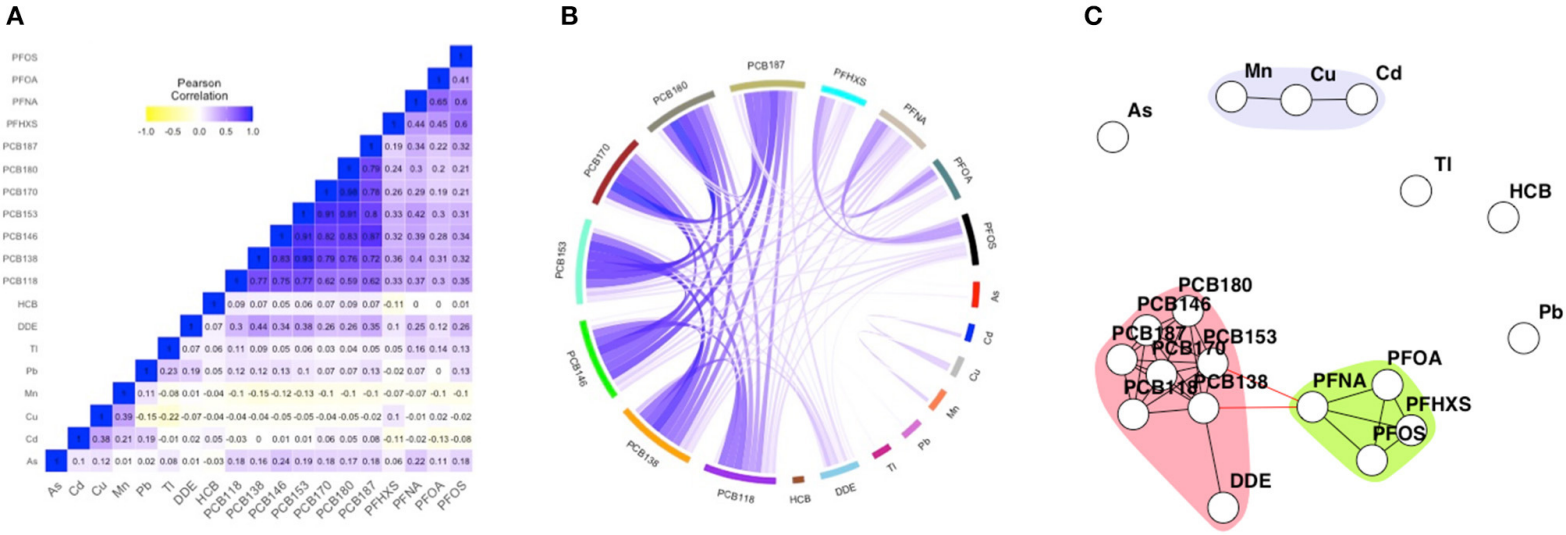
Heatmap **(A)**, circular correlation globe **(B)** and network including community detection **(C)** of FLEHS III, 19 biomarkers, *n* = 281. Data is corrected for maternal age, smoking during pregnancy and maternal pre-pregnancy BMI. The heatmap is based on Pearson correlation between the biomarkers. Within the circular globe each biomarker is presented as a color-block on the circular axis. Within the network, each dot or node represents a biomarker, each edge represents a connection between the biomarkers, each different color represents a community within a subnetwork.

Concentrations of biomarkers were natural log transformed because distributions were skewed. *p,p*′-DDE, HCB, and PCB concentrations were expressed as concentrations per gram blood lipid and as such corrected for differences in dietary fat intake. Hence it is expected that the correlations are independent of blood fat levels (20). Biomarker values below LOD were imputed based on a maximum likelihood estimation via single conditional imputation, dependent on observed values for the other biomarkers (21). Missing values in biomarkers and determinants (cholesterol, maternal age, maternal pre-pregnancy BMI, parity, singleton or multiples, and maternal smoking during pregnancy) were imputed by using a single imputation strategy stratified per campaign, using the R package *mice*. Determinants were imputed first, using linear regression for continuous variables, and logistic regression for the binary variables. The determinants and observed values were then used as prediction matrix for single imputation of the biomarkers (completely missing, e.g., due to insufficient blood volume), using linear regression. The geometric mean, minimum and maximum (based on imputed data) biomarker concentrations, and percentage of missing samples are presented in Supplementary Table 2. Pearson correlation structures between the natural logarithm transformed biomarkers per sampling campaign are presented by heatmaps and circos plots in Figure 1 and the Supplementary Figures 1, 2.

For comparisons across sampling campaigns, analytical datasets were created in which biomarker concentrations were residualized using a linear model incorporating predictors for maternal age, pre-pregnancy BMI and maternal smoking during pregnancy, following the corrections described by Schoeters et al. (16). For comparisons across covariate categories of smoking and BMI, analytical datasets of FLEHS III were created. The datasets stratified by smoking were adjusted for maternal age and maternal pre-pregnancy BMI; the datasets of BMI strata for maternal age and smoking during pregnancy.

### Network Graph Estimation and Community Detection

We used undirected and unweighted network analysis to describe the conditional independence between multiple variables, making use of the packages *huge* and *igraph*, using R (v3.5.0) (22, 23). A node in the network represents a biomarker, and an edge reflects conditional dependency given all other variables (23). For comparison purposes, weighted network analysis was applied as well, making use of the package *EGAnet (v0.9.6)* (24).

The graph estimation was conducted using the graphical lasso, which involves penalized maximum likelihood estimation (25). This method is a simple and fast algorithm for estimation of a sparse inverse covariance matrix using an L_1_ penalty. The graphical lasso cycles through the variables, fitting a modified lasso regression to each variable in turn. Regularization of the graph was conducted along a sequence of 10 equally spaced lambdas ranging from the maximum lambda (resulting in an empty graph) to the minimum lambda set at 10% of the maximum lambda. Optimal lambda selection was conducted using the stability approach to regularization selection method (StARS) (26), which selects the optimal lambda by variability across subsamples (26). Variability (or instability) across subsamples is defined as the fraction of times (range: 0–0.5) that two graphs disagree on the presence of an edge, averaged over all edges in the graphs. We used the default variability threshold of 0.1. Within the selected network, the *walktrap* algorithm from the *igraph* package was used, which performs random walks (in default of 4 steps) across the network to merge separate communities in a bottom-up manner (27, 28). Nodes were colored according to the community they were assigned to. Sensitivity analysis was performed by comparing the networks with and without inclusion of the *mice* imputed values (samples missing at random, see Supplementary Table 2 for percentages of missing's).

The low-dimensional setting of the FLEHS data also allows for the application of correlation networks (29). We compared our approach to an application of weighted correlation networks for the data in FLEHS I, II, and III. Weighted networks were estimated by the *EGA*net package, this Exploratory Graph Analysis technique was based on the Graphical lasso model and an EBIC tuning parameter of 0.5 was used (24, 30, 31). A parametric bootstrap (1,000 iterations) was used to estimate the median network structure. Communities in the EGA network were estimated using the *walktrap* algorithm. The weighted network shows the strength of the edge (absolute correlation) by thickness of the line, and direction of the correlation by color of the line (green for a positive correlation, red for a negative correlation).

Networks were constructed for each measurement campaign separately. Secondly, networks were constructed for different strata of the dataset of FLEHS III. Where FLEHS III was either split by maternal smoking status during pregnancy (yes n=33; no n=248), or by maternal pre-pregnancy BMI category (≤25 kg/m^2^ or low-normal *n* = 195; >25 kg/m^2^ or high *n* = 86).

### Comparative Network Analysis

Systematically comparing networks, or CNA, is of interest to assess the impact of covariates on networks derived in HBM data. Networks can be compared on their similarities or their dissimilarities. Multiple network comparison methods have been described before, and some can be computationally challenging (9, 32). In this paper, we focus on exact graph matching, which involves the exact correspondence between two or more graphs with the exact same set of nodes. We call an edge “conserved” if it is present in all of the input graphs. The complement of conserved edges is represented in a network graph (network of conserved edges). Comparative network analysis can also assess the presence of edges in network B which are not present in network A. These results can be interpreted as “additional” or different edges, and are presented in a network graph as well (network of differential edges). The CNA as applied in this paper focuses on differences in network structure, and not on differences in the detected communities. To assess the stability of the independently derived networks across the FLEHS sampling campaigns we conducted CNA to identify the conserved edges between the networks across campaigns. To evaluate the influence of covariates, differences between derived networks were assessed between the strata: high vs. low-normal maternal pre-pregnancy BMI, and non-smoking vs. smoking during pregnancy. Within the deduced conserved and differential networks multiple subnetworks were distinguished, within which the *walktrap* algorithm was applied for community detection (only if the subnetwork consisted of 6 or more nodes).

## Results

The study population, summary statistics and time trends of individual biomarkers over the three sampling campaign have been described previously (13, 16). An overview of the study characteristics and the concentrations per biomarker are presented in Supplementary Tables 1, 2.

The FLEHS III dataset consists of 19 biomarkers, and has been used to illustrate the network techniques since it is most data rich. Figure 1 presents the heatmap, correlation globe and network for the FLEHS III dataset. For comparison purposes, we present two alternative approaches to represent correlation structures in HBM data. In both the heatmap (Figure 1A) and the circular correlation globe (Figure 1B) correlation structures become apparent. The identification of communities of strongly correlated markers using these visualizations is not straight forward as it depends largely on the subjective interpretation of the reader. The heatmaps and correlation globes for FLEHS I and II are presented in Supplementary Figures 1A,B, 2A,B.

### Network Estimation and Community Detection

In the obtained network for FLEHS III, three communities were estimated. The markers of HCB, arsenic, thallium and lead were not part of a community. A subnetwork consisted of two connected communities, one with PCBs and *p,p*′*-*DDE, and one with PFAS (PFOA, PFOS, PFHXS and PFNA). The link between the two communities, marker PFNA within the PFOA community, was connected to PCB138 and PCB153. The other community consisted of cadmium, copper and manganese; and was not connected to any other communities. When we compare these networks to weighted networks derived in the same data (Supplementary Figure 4C), we observe the same communities of PCBs, PFAS and the metals cadmium, copper and manganese. Additionally, the metals thallium and lead also form a community. The markers for HCB and arsenic remain not part of any community.

The networks of FLEHS I and II are presented in the Supplementary Figures 1C, 2C. In the network for FLEHS I two subnetworks were estimated, one consisting of cadmium and lead, and the other consisting of PCB138/153/180, HCB and *p,p*′-DDE. In the network for FLEHS II four subnetworks were found, of which two were equal to FLEHS III (PCBs and PFAS). The community of the metals cadmium and lead was equal to FLEHS I. The weighted network for FLEHS I (including *walktrap* community detection algorithm) the community for the metals as the unweighted network (Supplementary Figure 4A). The markers for *p,p*′*-*DDE and HCB were estimated as a separate community, connected to PCB138/153/180. It can be seen that between the latter two communities the edges were strong. Within the weighted network for FLEHS II the exact same communities as the unweighted network were estimated (Supplementary Figure 4B). Sensitivity analysis was performed by comparing the networks with and without inclusion of the imputed values. No differences between those networks were found.

### Comparative Network Analysis

#### Differential Networks (Smoking During Pregnancy)

**Figure 2:**
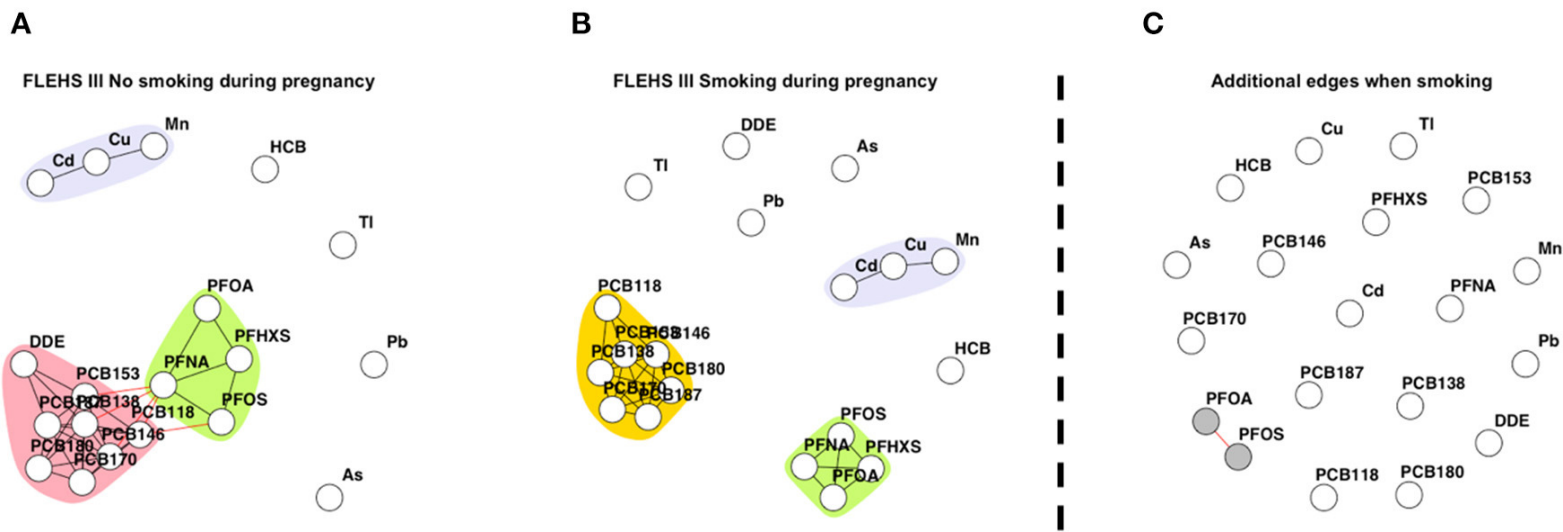
**(A)** Network based on individuals FLEHS III where the mother did not smoke during pregnancy (*n* = 248), **(B)** and mothers who smoked during pregnancy (*n* = 33). Within both **(A)** and **(B)** networks, each dot or node represents a biomarker, each edge represents a connection between the biomarkers, the different colors represent a community within a subnetwork. **(C)** Results of the CNA, dissimilar, or additional, edges when the mother smoked during pregnancy, only nodes part of a subnetwork are colored in gray. Data is corrected for maternal age and maternal pre-pregnancy BMI.

Figures 2A,B presents the networks consisting of biomarkers collected during FLEHS III, stratified by smoking status during pregnancy. Two hundred and forty-eight mothers did not smoke during pregnancy and 33 mothers did smoke during pregnancy. Equal to the total FLEHS III dataset, two subnetworks were identified for mothers who did not smoke during pregnancy. The graph of non-smoking mothers only differed by the connection of the community PCBs with PFAS, PFOS was also linked with the PCB community (Figure 2A). When the mother did smoke during pregnancy, three subnetworks were distinguished, one consisting of PCBs without *p,p*′-DDE, one of PFAs, and one with cadmium, copper and manganese (Figure 2B). Compared to Figure 2A and the network for the total FLEHS III dataset, the network of mothers who smoked had no connection between PCBs and PFAS. The results from the CNA presented in Figure 2C show one small subnetwork (colored in gray), reflecting the change in connection between PFOS and PFOA, that were not connected when the mother did not smoke during pregnancy, while they were connected when the mother did smoke. The CNA of the edges only present when the mother did not smoke during pregnancy are shown in Supplementary Figure 5. Here multiple edges between PFNA and PCBs, PFOS with PCB118, and *p,p*′-DDE with multiple PCBs were shown to be only estimated within the network of mothers who did not smoke during pregnancy.

#### Differential Networks (Maternal Pre-pregnancy BMI)

**Figure 3:**
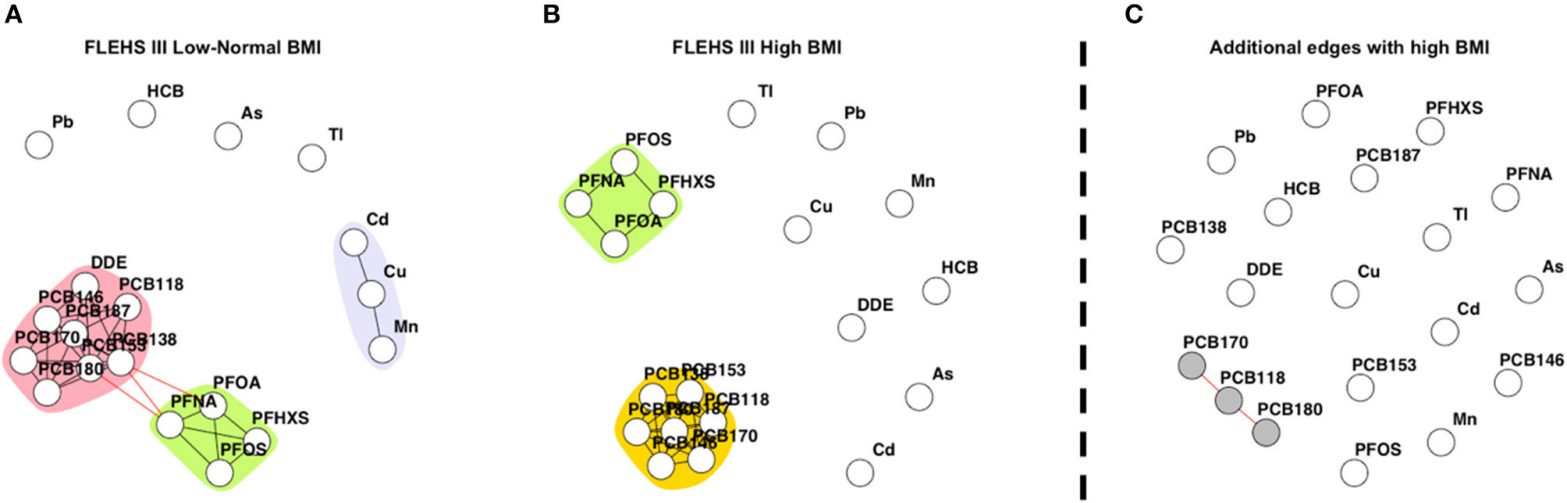
**(A)** Network based on individuals from FLEHS III split by low-normal maternal pre-pregnancy BMI (BMI ≤ 25 kg/m^2^, *n* = 195), **(B)** and high maternal pre-pregnancy BMI (BMI > 25 kg/m^2^, *n* = 86). Within both **(A)** and **(B)** networks, each dot or node represents a biomarker, each edge represents a connection between the biomarkers, the different colors represent a community within a subnetwork. **(C)** Results of the CNA, dissimilar, or additional, edges when the mother had a high BMI, only nodes part of a subnetwork are colored in gray. Data is corrected for maternal age and smoking status during pregnancy.

Figure 3 presents networks consisting of biomarkers collected during FLEHS III, stratified by maternal pre-pregnancy BMI. One hundred and ninety-five mothers had a low-normal pre-pregnancy BMI, and 86 mothers a high pre-pregnancy BMI. Within the network of the stratum of mothers with a low-normal pre-pregnancy BMI (≤25 kg/m^2^), two subnetworks were identified. The detected subnetworks and communities were the same as in the total FLEHS III dataset. The PCB community was connected to PFAS, and the community of cadmium/copper/manganese was not connected to any other (Figure 3A). Within the stratum of mothers with a high pre-pregnancy BMI (>25 kg/m^2^) only communities for PCBs and PFAS were estimated, which were not connected (Figure 3B). Also, *p,p*′*-*DDE was not part of the PCB community. Comparative network analysis of the networks, presented in Figure 3C, shows the dissimilar edges between the strata. The edges additional to the network for mothers with high pre-pregnancy BMI were identified and colored in gray: PCB118, PCB170, and PCB180. The CNA results showing edges only present for mothers with low-normal BMI are shown in Supplementary Figure 5. Multiple edges between DDE and PCBs were estimated, as well as the edges between manganese, copper and cadmium.

#### Conserved Networks Across Campaigns

**Figure 4:**
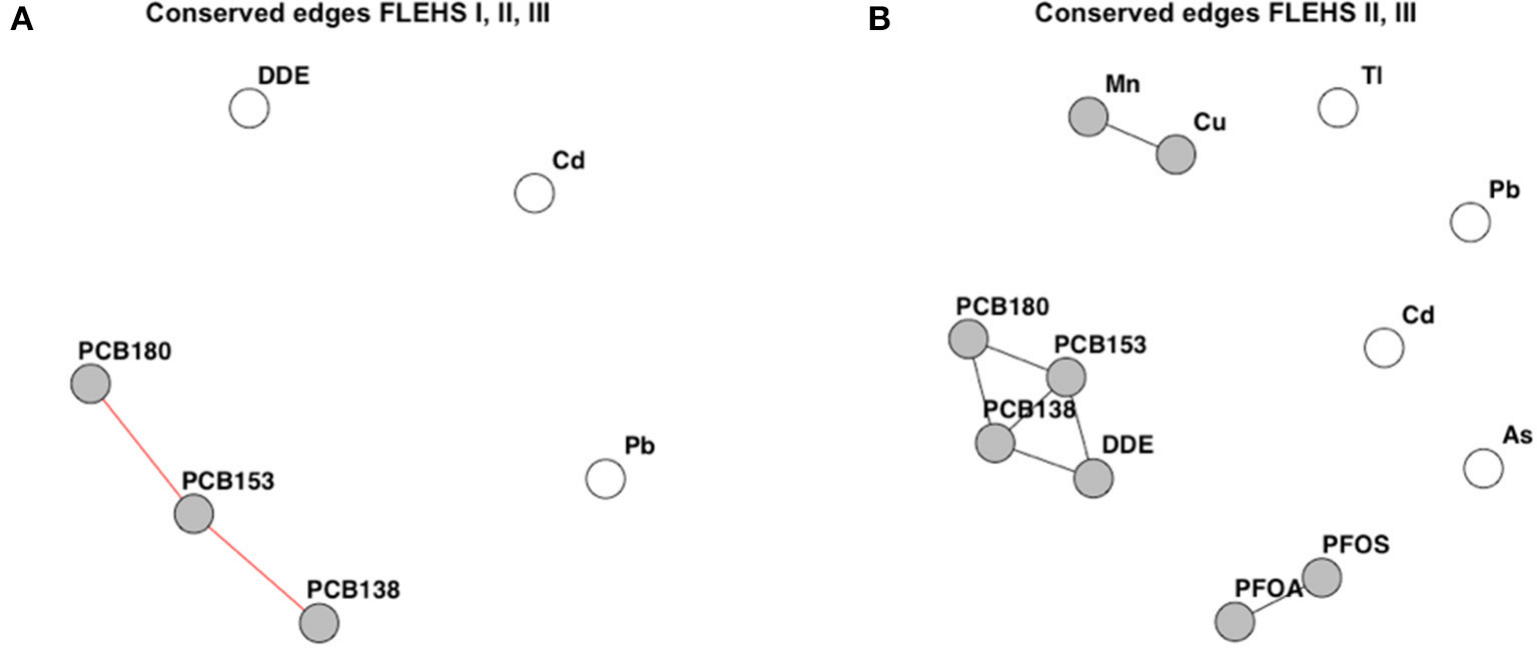
Results of the CNA across three campaigns **(A)**, or between two campaigns **(B)**. Resulting networks are the similar edges, present in either all three, or both, of the networks per FLEHS campaign. **(A)** Conserved or similar edges over all three networks of FLEHS I, II and III, based on six biomarkers. **(B)** Conserved or similar edges between the two networks of FLEHS II and III, based on 12 biomarkers. Conserved edges are presented as a network graph, only nodes part of a subnetwork are colored in gray.

Figure 4A presents the conserved edges across the three networks that were independently derived in the FLEHS I, II, and III datasets (containing the 6 biomarkers measured in all three campaigns). The individual networks derived on the six biomarkers measured in FLEHS I, II, and III are presented in the Supplementary Figure 3. Edges between PCB138, PCB153, and PCB180 were seen in all three campaigns. *p,p*′*-*DDE, lead and cadmium were not included as a subnetwork of this CNA, as these were not consistently correlated across the three campaigns. Figure 4B presents the conserved edges based on FLEHS II, and III datasets (containing the 12 biomarkers measured in both campaigns). Here, three subnetworks were identified: PFOA and PFOS; *p,p*′-DDE and PCB138/153/180; manganese and copper. These subnetworks identified are the biomarkers that were consistently connected in both sampling campaigns. Arsenic, cadmium, thallium and lead were not included in any of the subnetworks, and therefore not connected to the same biomarkers in both FLEHS II and FLEHS III networks.

## Discussion

We provide an application of network analysis in HBM data. The primary utility of this work is to demonstrate that network methodologies can be used to identify prevalent mixtures of chemicals in HBM data. Conditional independence networks provide a data-driven and intuitive approach to highlight the presence of highly connected biomarker measurements without prior assumptions or groupings, about for example sources, chemical properties, pathways or mode of actions. The primary benefit of a network over the heatmap or circos plots is the ease of identification, formalization of the procedure to identify communities and providing a structural approach for comparison of exposure patterns between datasets or across strata within the dataset.

At the same time, some information is potentially lost when describing an HBM dataset using conditional independence networks. Heatmaps and circos plots provide information on the degree of correlation. As such the applied network methodology is an addition to other graphical presentations, not a replacement. The networks as described in the Results section are based on unweighted edges, which become of more value in high dimensional HBM data such as untargeted screening data. Weighted partial correlation networks that include information on the degree and direction of association between biomarkers, can provide additional information especially when the number of nodes is not too large and a visual interpretation can be made (31). In addition to graphical tools, approaches such as principal components or cluster analysis (33) can provide insight into complex correlation structures in the data, but are often more difficult to digest visually, especially in high dimensional settings.

Network techniques can be used as a first screening technique to assess patterns in mixture exposure biomarker data and comparisons across strata of covariates, to assist exposure scientists (pathway, source identification), to assist epidemiologists in taking the communities into account during data analysis and interpretation, and to guide toxicological mixture experiments in identifying real-life mixtures.

### Worked Example: FLEHS Datasets

The application in FLEHS provided some examples of insights that can be acquired by applying networks in HBM data. The community structures we detected in the FLEHS data are in line with earlier findings that groups with similar chemical structures such as PCBs group together (34). As expected based on previous analyses and literature, due to their often observed high correlation structure, we observed a PCB community in all derived networks, which could be explained by shared sources and similar kinetics (33, 35, 36). We also note, however, that sometimes biomarker *p,p*′*-*DDE was included in the “PCB community” highlighting that, when assessing the impact of PCBs, one potentially needs to take into account concurrent exposure of *p,p*′*-*DDE. This was also observed in a previous analysis of the FLEHS data (37), where an association between *p,p*′-DDE and birth weight was observed while correcting for PCBs, which was not observed in a single pollutant model between *p,p*′-DDE and birth weight. Such findings underline that assessing health risks of combinations of exposure biomarkers reflects better real-world situations and thereby allow more effective risk assessment. Another group of typically highly correlated compounds, the PFAS, were consistently identified as a community in our networks. For the metals the size and composition of the communities varied across the FLEHS campaigns, likely reflecting rather dispersed sources of metal exposure. Within some of the networks, some biomarkers were not included in a subnetwork (such as HCB in FLEHS III), which could be expected since the partial correlation with other biomarkers was very low (no links to other markers in the circos plot), indicating different exposure sources and/or kinetics.

The results of the CNA between the three datasets (Figure 4A), show that the association of the PCBs with *p,p*′*-*DDE is not always based on the same PCB, and therefore doesn't show as a conserved link across all three campaigns. Multiple explanations can be hypothesized, such as a change in correlation between source and usage over time, causing a change in correlation. Also, the concentration of DDT/DDE/DDD changes over time (e.g., by regulation), as well as the composition of the PCB mixture. The smaller number of samples analyzed in FLEHS II and FLEHS III might also mean that there is a larger impact of random variation or error in the estimated networks, which would explain the observed variation as well.

As an example, the FLEHS data was stratified by smoking status and pre-pregnancy BMI, other strata such as diet (e.g., fish consumption) are also possible. The FLEHS III networks stratified by smoking, both had an equal composition of communities. With the difference that when the mother smoked during pregnancy, an additional edge between PFOA/PFOS was estimated. We could not identify a straightforward explanation for this observation, yet potential explanations would include metabolic changes due to smoking behavior, or a co-exposure that occurs only with smoking women (38). Moreover, only 33 mothers indicated they smoked during pregnancy, which could indicate reduced statistical power to detect true correlations. Also, since this variable indicates if they have ever smoked during pregnancy it could be that the actual smoking frequency was rather low as mothers would be aware of the bad influence of smoking on their unborn child. In the network derived in mothers with high pre-pregnancy BMI we see that the biomarkers form two communities, one with all PCBs and one with PFAS. While both communities were connected when the mother had a low-normal BMI, which could be explained in differences in diet or other lifestyle factors. The results of the CNA between smoking and BMI such as in Figures 2C, 3C give direction in thinking about common exposure sources or common exposures due to lifestyle factors (e.g., dietary habits, low SES, smoking) that contribute to the correlation patterns in HBM exposure biomarkers and will help to prioritize concurrent exposures that could be considered together when assessing exposure-effect associations.

In a biomonitoring study with relatively limited number of markers measured, such as the FLEHS campaigns, weighted networks can be applied as well. In our application a weighted network provided similar insights to our conditional independence method: communities overlap between both methods. The only difference in community was thallium and lead in the FLEHS III dataset, which had the weakest within-community edge. Most likely the detection of this community is just above the threshold; it is dropped in the unweighted network as a result of the slightly different network estimation. In the weighted networks the edges that connect the communities are clearly less present (thinner) or not present at all. As such there was no significant loss of information by choosing for an unweighted network method. In high dimensional settings the application of weighted networks might become unwieldy and therefore we suggest our method in such settings.

### Limitations

There were several limitations to the application of the network analysis in the FLEHS data. First of all, this work is based on a limited set of biomarkers, which reduced the added information of the network estimation, but on the other hand presented easily interpretable networks. Due to the limited number of biomarkers in FLEHS I and II, it was decided to focus the stratification by covariates only on the FLEHS III dataset with 19 biomarkers. Secondly, the amount of observations was limited. For the comparisons of BMI category or smoking status the amount of observations in one of the strata was limited (minimum of *n* = 33). Thirdly, an underlying assumption of the temporal comparisons between the FLEHS campaigns, is the comparability between the campaigns. Analysis of the biomarkers was done by the same lab in the subsequent campaigns, and control samples were analyzed to assess the comparability of the results. However, different individuals were measured in the different campaigns and slight variations in demographics between participants by campaign could result in different networks.

### Future Extensions

While not opportune in our current dataset, further extensions to the currently described methods can be foreseen. For example, rather than focusing on differences in networks across covariates, one could focus on differences in communities: Differential Community Detection (12). Since the amount of different communities per network was limited for the FLEHS data, this would not have added much information in the FLEHS datasets, but would in high dimensional HBM datasets. Also, the focus on the community differences would be important for applications in epidemiology, mixture toxicology, and mixture risk assessment. The communities in a network can be considered as starting points for further assessment of mixture health effects or in the design of mixture toxicology studies, providing information on combined exposures that occur at population level. Mixture risk assessment might indirectly use the community information, focusing on a common health effect for all substances in the community. Depending on the risk assessment purpose, it might be of use to apply overlapping community detection, where one biomarker could be part of multiple communities (fuzzy clustering) (39).

The application of weighted correlation networks to the FLEHS data did not yield substantially differing insights as compared to the results obtained with the application of the conditional independence methods. This is likely explained by the strong communities that exist in this data and that the correlation matrix is largely positive. However, in other datasets of similar dimensions, weighted network approaches can be a useful addition by providing more information (degree and direction) on the associations between biomarkers, underlying the observed communities. When the number of biomarkers in the dataset increases, the interpretation of weighted networks is likely to become more challenging, although community detection will facilitate interpretation to a great deal. Also, CNA of weighted networks will become more challenging, for example inexact graph matching where networks are assessed as equal within certain criteria (32), or where the most important nodes and/or edges are extracted (40).

The network approaches presented here will be a worthwhile tool when applied in high dimensional HBM datasets. Technological developments are making such datasets increasingly possible by application of methods such as untargeted high resolution mass spectrometry (41–43). The application of network analysis could help identifying clusters in the data, including parent compounds and related metabolites. Network analysis on high dimensional data has great potential for mixture risk assessment to describe the complex exposure patterns, their composition and variability. Comparative network analysis on strata of covariates may identify specific risk groups with particular communities of biomarkers of concern. While initial steps have been made toward the risk assessment of mixtures, these approaches are often either based on the assessment of chemically related compounds (e.g., PCB congeners), or based on toxicology (44–46), and not on common occurrence and exposure patterns. Insights into complex correlation networks in HBM data, and the presence of communities within these networks, provide useful information on the presence of mixtures at population level.

## Data Availability Statement

The data analyzed in this study is subject to the following licenses/restrictions: Access to the data can only be granted by the data owner. Requests to access these datasets should be directed to TZC@provincieantwerpen.be.

## Ethics Statement

The studies involving human participants were reviewed and approved by Ethical Committee of the University of Antwerp (FLEHS I and II) and of the University Hospital of Antwerp (FLEHS III). The patients/participants provided their written informed consent to participate in this study.

## Author Contributions

IO participated in the design of this research, performed the statistical analysis, and writing the manuscript. EG contributed to the data collection, data analysis, and writing the manuscript. IO and EG had an equal contribution to this manuscript. JV conceived and designed the research, participated in data analysis, and writing the manuscript. GS, EL, and RV contributed to the design of this research, provided feedback on the statistical analyses, and assisted in writing the manuscript. All authors reviewed and approved the final version of the manuscript.

## Conflict of Interest

The authors declare that the research was conducted in the absence of any commercial or financial relationships that could be construed as a potential conflict of interest. The reviewer RK declared a past co-authorship with one of the authors RV to the handling Editor.

## Abbreviations

CNA: Comparative Network Analysis
EGA: Exploratory Graph Analysis
FLEHS: Flemish Environment and Health Study
HBM: Human Biomonitoring
HCB: Hexachlorobenzene
LOD: Limit of Detection
PCBs: Polychlorinated biphenyls
PFAS: Per- and polyfluoroalkyl substances
PFHXS: Perfluorohexane sulfonate
PFNA: Perfluorononanoic acid
PFOA: Perfluorooctanic acid
PFOS: Perfluorooctane sulfonate
POPs: Persistent Organic Pollutants
*p,p*′-DDE: *p,p*′-Dichlorodiphenyldichloroethylene.
