# Supplementary material for: Network Analysis to Identify Communities Among Multiple Exposure Biomarkers Measured at Birth in Three Flemish General Population Samples
Source: Front Public Health. 2021 Feb 10;9:590038. doi: 10.3389/fpubh.2021.590038 (PMC7902692; doi:10.3389/fpubh.2021.590038)
Supplement: Supplementary file 1 [file Table_1.docx]

Table S1. Characteristics of the FLEHS newborn campaigns: mean (min-max). P-value was calculated by ANOVA for continuous variables, and by Chi-square for categorical outcomes.

| **Mothers** | **FLEHS I** | **FLEHS II** | **FLEHS III** | **p-value** |
| --- | --- | --- | --- | --- |
| Number of participants | 1196 | 255 | 281 |  |
| Age (years) | 29.6 (18.1 – 44.0) | 30.3 (18.2 – 42.4) | 30.2 (18.9 – 44.8) | 0.02 |
| Mean pre-pregnancy BMI (kg/m^2^) | 23.3 (14.0 – 44.6) | 23.5 (16.0 – 47.4) | 23.9 (15.2 – 45.2) | 0.13 |
| Mean duration of pregnancy (weeks) | 39 (31-42) | 39 (34-42) | 39 (35-42) | 0.52 |
| Primipari (%) | 60.8 | 39.8 | 44.8 | <0.0001 |
| Smoking during pregnancy (%) | 16.2 | 11.6 | 11.7 | 0.06 |
| Never drinking alcohol during pregnancy (%) | 91.7 | 55.5 | 74.7 | <0.0001 |
