# Supplementary material for: Network Analysis to Identify Communities Among Multiple Exposure Biomarkers Measured at Birth in Three Flemish General Population Samples
Source: Front Public Health. 2021 Feb 10;9:590038. doi: 10.3389/fpubh.2021.590038 (PMC7902692; doi:10.3389/fpubh.2021.590038)
Supplement: Supplementary file 2 [file Table_2.docx]

Table S2. Geometric mean exposure concentrations of the biomarkers, based on the data after imputation and fat-correction, split by measurement campaign. As well as the percentage of missing values per biomarker, the % missing are missing at random, e.g. due to laboratory sample loss or insufficient blood volume.

| **Variable (unit)**  **/ Campaign** | **FLEHS I** | | **FLEHS II** | | **FLEHS III** | |
| --- | --- | --- | --- | --- | --- | --- |
|  | **Geomean  (Min - Max)** | **% missing** | **Geomean  (Min-Max)** | **% missing** | **Geomean  (Min-Max)** | **% missing** |
| Arsenic (As)  (μg/L) |  |  | 0.55 (0.03;14.40) | 2.35 % | 0.71 (0.09;19.00) | 0 % |
| Cadmium (Cd)  (μg/L) | 0.19 (0.00;13.87) | 7.02 % | 0.08 (0.01;5.31) | 2.35 % | 0.02 (0.01;0.10) | 0 % |
| Copper (Cu)  (μg/L) |  |  | 598.46 (299.0;994.0) | 2.35 % | 556.79 (310.0;995.0) | 0 % |
| Manganese (Mn)  (μg/L) |  |  | 31.46 (7.29;80.12) | 2.35 % | 30.10 (12.46;81.05) | 0 % |
| Lead (Pb)  (μg/L) | 13.29 (0.63;177.61) | 7.36 % | 8.60 (2.40;67.40) | 2.35 % | 6.44 (1.93;43.92) | 0 % |
| Thallium (Tl)  (ng/L) |  |  | 16.89 (8.00;41.00) | 2.35 % | 18.59 (8.73;43.86) | 0 % |
| p.p’-DDE  (ng/g lipid) | 108.16 (6.64;1815.53) | 6.86 % | 77.04 (9.76;641.08) | 0.78 % | 59.92 (8.85;903.18) | 1.78 % |
| HCB  (ng/g lipid) | 17.63  (0.94;402.07) | 12.46 % |  |  | 11.61 (1.26;72.07) | 1.78 % |
| PCB118  (ng/g lipid) |  |  |  |  | 3.36 (0.33;15.26) | 1.78 % |
| PCB138 (ng/g lipid) | 14.68 (0.56;156.86) | 11.62 % | 17.20 (1.68;69.78) | 0.78 % | 10.31 (1.83;39.68) | 1.78 % |
| PCB146  (ng/g lipid) |  |  |  |  | 1.49 (0.24;6.88) | 1.78 % |
| PCB153  (ng/g lipid) | 25.69 (0.93;230.21) | 10.70 % | 26.77 (4.78;108.89) | 0.78 % | 16.50 (2.91;53.97) | 1.78 % |
| PCB170  (ng/g lipid) |  |  |  |  | 4.34 (0.57;20.18) | 1.78 % |
| PCB180  (ng/g lipid) | 20.41 (1.51;153.13) | 10.20 % | 15.64 (2.68;70.18) | 0.78 % | 8.57 (0.99;56.42) | 1.78 % |
| PCB187  (ng/g lipid) |  |  |  |  | 2.39 (0.40;21.10) | 1.78 % |
| PFHXS  (μg/L) |  |  |  |  | 0.36 (0.06;1.33) | 4.27 % |
| PFNA  (μg/L) |  |  |  |  | 0.21 (0.05;1.39) | 4.27 % |
| PFOA  (μg/L) |  |  | 1.53 (0.50;4.30) | 13.73 % | 1.19 (0.26;5.87) | 4.27 % |
| PFOS  (μg/L) |  |  | 2.69 (0.80;17.30) | 13.73 % | 1.11 (0.13;8.37) | 4.27 % |
