# Supplementary figures and images for: Network Analysis to Identify Communities Among Multiple Exposure Biomarkers Measured at Birth in Three Flemish General Population Samples
Source: Front Public Health. 2021 Feb 10;9:590038. doi: 10.3389/fpubh.2021.590038 (PMC7902692; doi:10.3389/fpubh.2021.590038)

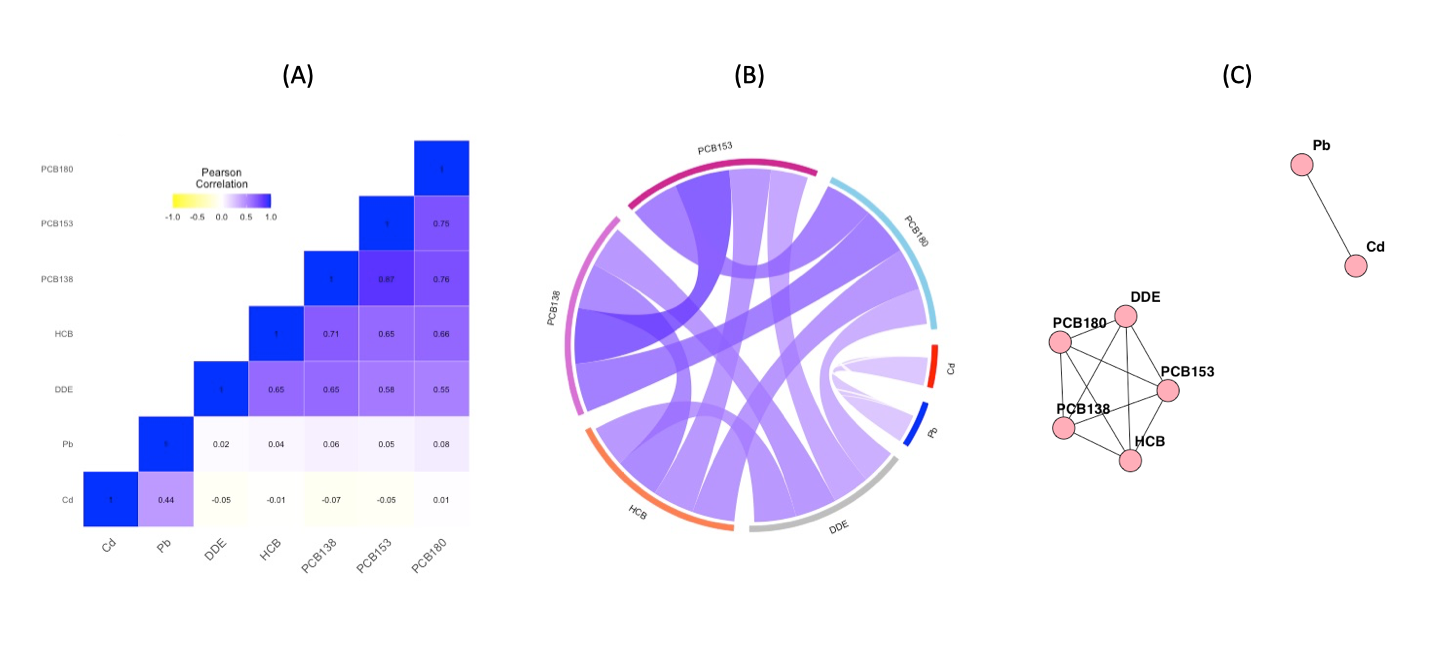

Supplement: Supplementary file 3 [file Image_1.TIFF]

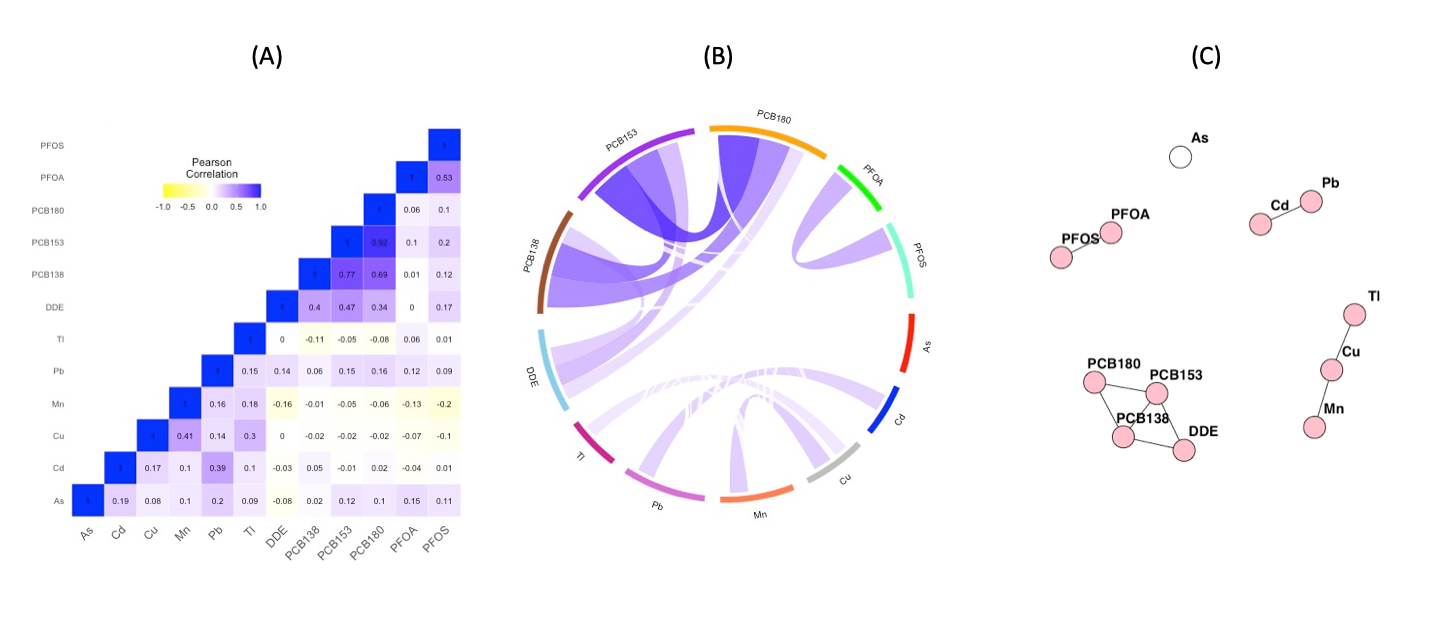

Supplement: Supplementary file 4 [file Image_2.TIFF]

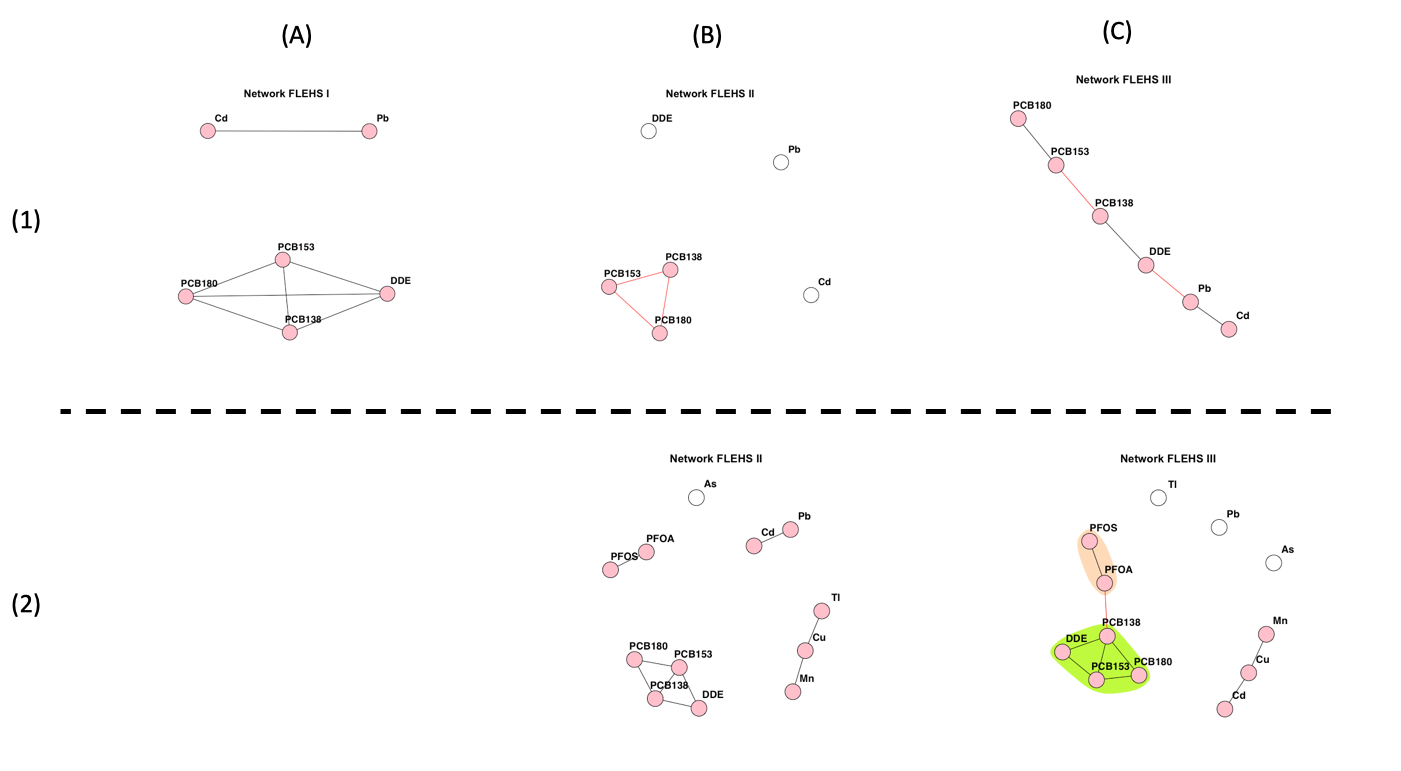

Supplement: Supplementary file 5 [file Image_3.TIFF]

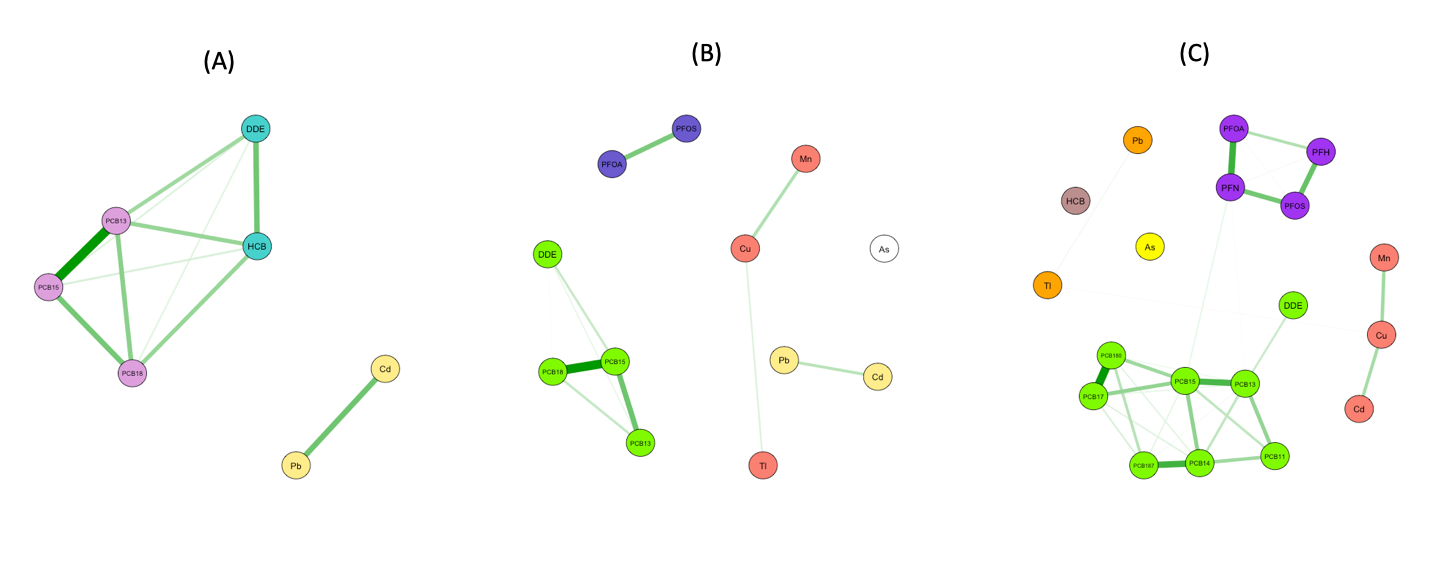

Supplement: Supplementary file 6 [file Image_4.TIFF]

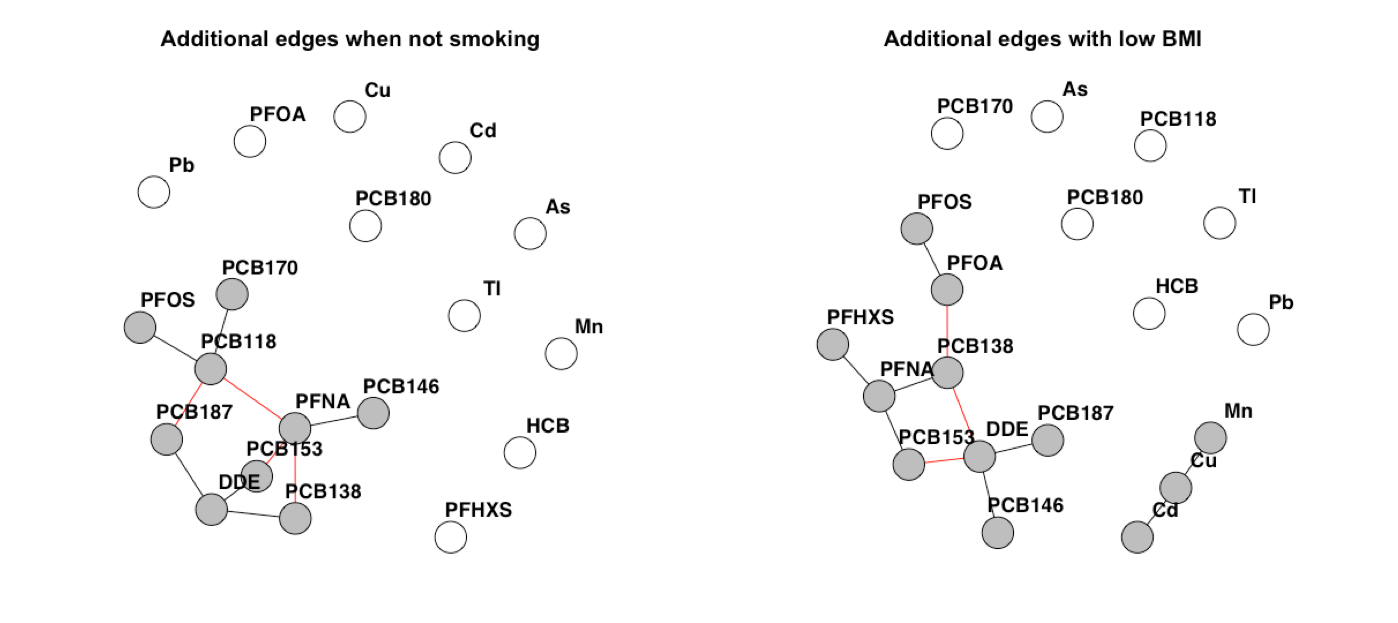

Supplement: Supplementary file 7 [file Image_5.TIFF]
